# Genome-Wide Identification and Expression Analysis of *Calmodulin* (CaM) and *Calmodulin-Like* (CML) Genes in the Brown Algae *Saccharina japonica*

**DOI:** 10.3390/plants12101934

**Published:** 2023-05-09

**Authors:** Nianchao Xue, Minghui Sun, Zihan Gai, Meihan Bai, Juan Sun, Shan Sai, Linan Zhang

**Affiliations:** 1 School of Marine Science and Engineering, Qingdao Agricultural University, Qingdao 266109, China; 2 National Engineering Science Research & Development Center of Algae and Sea Cucumbers of China, Shandong Technology Innovation Center of Algae and Sea Cucumber, Provincial Key Laboratory of Genetic Improvement & Efficient Culture of Marine Algae of Shandong, Shandong Oriental Ocean Sci-Tech Co., Ltd., Yantai 264003, China

**Keywords:** brown algae, *Saccharina japonica*, calmodulins, calmodulin-like protein, gametophyte development

## Abstract

Calmodulins (CaMs) and Calmodulin-like proteins (CMLs) are vital in plant growth, development, and stress responses. However, CaMs and CMLs have not been fully identified and characterized in brown algae, which has been evolving independently of the well-studied green plant lineage. In this study, whole-genome searches revealed one SjCaM and eight SjCMLs in *Saccharina japonica*, and one EsCaM and eleven EsCMLs in *Ectocarpus* sp. SjCaM and EsCaM encoded identical protein products and shared 88.59–89.93% amino acid identities with *Arabidopsis thaliana* AtCaMs, thereby indicating that brown algae CaMs retained a similar Ca^2+^ sensors function as in plants. The phylogenetic and gene structure analysis results showed that there was significant divergence in the gene sequences among brown algae CMLs. Furthermore, evolutionary analysis indicated that the function of brown alga CMLs was relatively conserved, which may be related to the fact that brown algae do not need to face complex environments like terrestrial plants. Regulatory elements prediction and the expression analysis revealed the probable functioning of SjCaM/CML genes in gametophyte development and the stress response in *S. japonica*. In addition, the SjCaM/SjCMLs interacting proteins and chemicals were preliminarily predicted, suggesting that SjCaM/SjCMLs might play putative roles in Ca^2+^/CaM-mediated growth and development processes and stimulus responses. Therefore, these results will facilitate our understanding of the evolution of brown algae CaMs/CMLs and the functional identification of SjCaM/SjCMLs.

## 1. Introduction

Calcium ions (Ca^2+^) are one of the most widely available and used second messengers in eukaryotic cells [1,2]. In growth and developmental processes and during abiotic and biotic stress conditions, rapid changes in Ca^2+^ levels in cells caused either by extracellular events or Ca^2+^ influx can trigger physiological changes and help coordinate adaptive responses. The Ca^2+^ signaling must be decoded, relayed, and amplified by Ca^2+^-binding proteins (Ca^2+^ sensors), which bind diverse downstream protein targets to carry out the appropriate responses [3,4]. Eukaryotes have evolved an arsenal of Ca^2+^-binding proteins, which usually contain multiple paired EF-hand motifs and a helix–loop–helix structure. They can be divided into different protein families, mainly including calmodulin (CaM), CaM-like protein (CML), calcium-dependent protein kinase (CDPK), and calcineurin B-like proteins (CBL) [5,6,7,8].

CaM is the most quintessential and ubiquitous Ca^2+^-sensing protein in eukaryotes [9,10]. It typically comprises 148 residues with four EF-hand motifs that change their conformation upon binding to Ca^2+^. Each EF-hand motif contains two alpha helices that are connected by a 12 amino acid residue loop. CaM was the most conserved during the evolution of the Ca^2+^-sensing protein. All known vertebrate CaMs are identical in amino acid sequence and share 91% amino acid identity with plant CaMs [11]. Similar to CaM, CML contains only the EF-hand domain and lacks any other functional domains or intrinsic activities. However, CML has only been found and characterized in plants and certain protist groups [5]. CML is more widely divergent than CaM at the structural level. It shows variations in EF-hand sequences and EF-hand motif numbers, even in a single species [12]. Over the past several years, the whole-genome sequencing of an increasing number of plant species has helped identify increasing CaM and CML family proteins in multiple plant species, including *Arabidopsis thaliana* [11], *Oryza sativa* [13], *Glycine max* [14], *Vitis vinifera* [15], *Brassica napus* [16], *Solanum pennelllii* [17], *Hordeum vulgare* [18], and *Triticum aestivum* [19], with their diverse and multiple functions being explored and analyzed worldwide. Increasing number of reports showed that CaM and CML genes are vital in plant growth and development [20]. In *Arabidopsis*, CaMs participate in brassinosteroid hormone biosynthesis and pollen development via their downstream targets, i.e., DWF1 and NPG1, respectively [21,22]. CMLs are also involved in multiple developmental processes, such as flowering, pollen germination and tube elongation, seedling development, and trichome branching [23,24,25,26]. Additionally, multiple studies supported the idea that CaMs, CMLs, and downstream elements are also important players in the disease and herbivore resistance and abiotic stress (e.g., salinity, temperature, and drought) response. Most *Arabidopsis* CaMs/CMLs are an integral part of the regulatory network of multiple signaling pathways of both abiotic and biotic stress tolerance, including CAM7 [27], CML8 [28,29], CML37 [30,31], and CML42 [32]. Recently, the evolution of CaMs and CMLs in different representative species of the green plant lineage has been published [12]. The expansion of the CaM and CML families from algae to land plants was supposed to be driven by selective pressure to enable the adaptation to varied terrestrial environments and the development of novel functions necessary for colonizing new habitats [12]. However, the identification of CaM and CML in other eukaryotic lineages except plants and animals is currently limited, and their functions are poorly understood.

Brown algae belong to the group Stramenopiles in the TSAR eukaryotic lineage, a eukaryotic branch highly divergent from animal, fungi, and green plant lineages [33]. In brown algae, the preliminary studies related to calcium and calcium-binding proteins were only found in the development process of fucoid algae. The cytoplasmic-free calcium gradient was detected in eggs and growing rhizoid cells [34,35]. Meanwhile, calmodulin and calmodulin-binding proteins were found to be involved in diverse physiological processes, including fertilization, zygotic photopolarization, and early embryonic development through biochemical analysis [36,37,38]. Recently, the whole genomes of multiple brown algae (*Ectocarpus*, *Saccharina*, *Cladosiphon*, and *Undaria*) have been reported, which facilitated the screening of the CaM and CML family of brown algae at the whole-genome scale [39,40,41,42,43]. In the previous studies, some calcium-binding-related proteins of brown algae were annotated and these showed differential expression patterns among different developmental stages and between the sexes [44,45]. However, the identification of CaMs and CMLs and their potential functions in brown algae still remain unclear.

*Ectocaropus* is a model brown algae system for studying developmental processes, life cycle, and evolution [46,47,48,49,50]. *Saccharina japonica* is the most commercially important kelp species in China [51]. In this study, CaM and CML genes were identified in *Ectocarpus* sp. and *S. japonica*, respectively. Their phylogenetic relationships, gene structures, selection pressure, *cis*-acting elements, and interacting proteins were analyzed subsequently. Furthermore, we conducted the expression pattern analysis of the SjCaM/SjCML genes using both RNA-seq and qRT-PCR data. Therefore, our findings not only provide a comprehensive analysis of the CaM/CMLs in brown algae but also facilitate further functional identification of SjCaM/SjCMLs in *S. japonica*.

## 2. Results

### 2.1. Identification of CaM/CML Genes in Brown Algae

**Figure 1:**
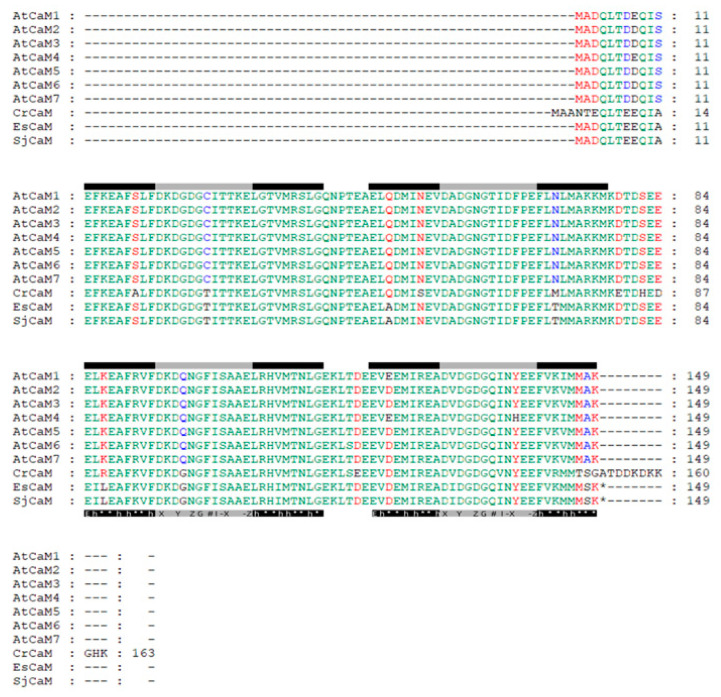
Alignments of amino acid sequences of the brown algae CaM family proteins with the green lineage. The regions corresponding to the E helices, the Ca^2+^-binding loops, and the F helices are indicated by the black, gray, and black bars, respectively. The consensus sequences for these regions are indicated beneath the relevant sequences. “E” stands for glutamic acid, “h” for hydrophobic amino acid, and “*” for any amino acid. “X, Y, Z, G, #, I, -X, -Z” are defined as follows: The X position is almost exclusively filled with aspartate (D); Y is usually aspartate (D) or asparagine (N); Z is aspartate (D), asparagine (N), or serine (S); the # position tolerates a variety of amino acids; -X also varies, but is usually aspartate (D), asparagine (N), or serine (S); -Z, which contributes two coordination sites, is nearly invariably glutamate ©. Glycine (G) at position 6 is highly conserved and is thought to provide the ability for a sharp turn within the loop. Position 8 is most often isoleucine (I), which can form hydrogen bonds with the other EF loop in a pair. The cystei©(C) residue in position 7 of the first EF hand is common among plant CaMs. Amino acid sequence identities are shaded. At, *Arabidopsis thaliana*; Cr, *Chlamydomonas reinhardtii*; Sj, *Saccharina japonica*; Es, *Ectocarpus* sp.

Using a combination of the results of BLASTP and HMMER research, we obtained a total of 26 and 35 CaM/CML proteins containing EF-hand domain in the *S. japonica* and *E.* sp. protein databases, respectively (Table S1). Then, we discarded those that contained domains other than the EF-hand domain and whose polypeptide length was significantly longer than CaMs. Finally, we designated the 9 and 12 filtered sequences as SjCaM/SjCMLs and EsCaM/EsCMLs, respectively (Table 1 and Table S2). Among them, we identified only one SjCaM (SJ01891) and one EsCaM (Ec-06_002270.1), and they shared 88.67% identity at the nucleotide level while encoding identical protein products. SjCaM/EsCaM had the same protein length (149 aa), number of EF-hands (4), and high degrees of amino acid identities (88.59–89.93%) as AtCaMs (Table S3). They also showed shared conserved amino acid residues and sequences-related functionality with plant CaMs, including lysine at position 116, six amino acids (at positions 1, 3, 5, 7, 9, and 12) serving as Ca^2+^-binding ligands in the Ca^2+^ binding loop in each EF-hand, and the distribution pattern with hydrophobic amino acids in both the E and F helices (Table 1; Figure 1). Additionally, we also detected a high proportion of methionine (~7.4%) in brown alga CaMs (Table 1).

We identified a total of 8 SjCMLs and 11 EsCMLs and named them in the order of their respective protein sequence identity to SjCaM/EsCaM (Table 1). All the CMLs shared >26% identity with SjCaM/EsCaM and contained at least two EF-hand domains. Over half the CMLs contained four EF-hand domains for each species. The protein length, pI, MW, and methionine percentage (M%) of SjCMLs ranged from 130 to 215 aa, 4.04 to 6.10, 14.60 to 23.47 kDa, and 2.2% to 6.9%, respectively. We found similar characteristics in EsCMLs. Their length, pI, MW, and M% ranged from 154 to 182 aa, 4.03 to 5.42, 17.13 to 20.39 kDa, and 2.9% to 5.5%, respectively. The subcellular location prediction indicated that the CaM proteins were localized in the cytoplasm and the CMLs proteins were localized in the cytoplasm, chloroplast, nucleus, mitochondrion, and peroxisome.

### 2.2. Phylogenetic and Gene Structure Analysis of the Brown Algae CaMs/CMLs

**Figure 2:**
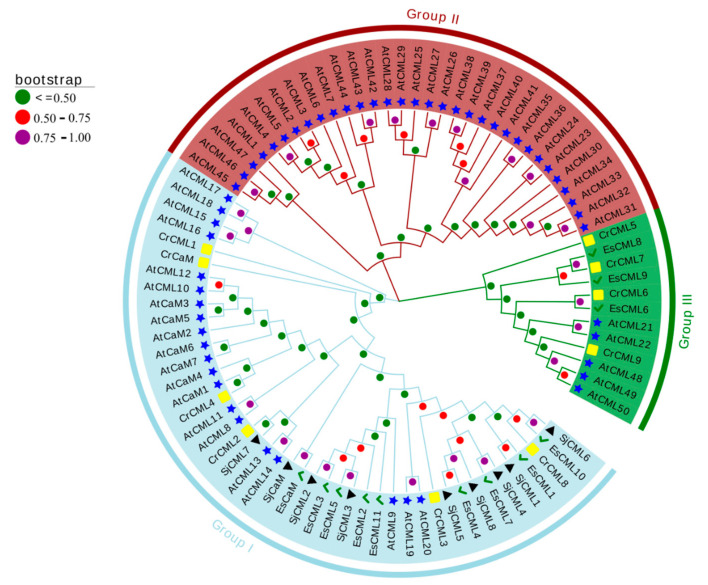
Phylogenetic analysis of the CaM/CML family of proteins amongst diverse species. The phylogenetic tree was constructed based on the sequence alignments by the ML method with bootstrapping analysis (1000 replicates) of the CaMs/CMLs proteins. The ML phylogenetic tree was created using ATGC PhyML 3.0. The proteins have been divided into three groups, each filled with a different color. Different colors and shapes represent different species. Black triangle, *Saccharina japonica*; green checkmark, *Ectocarpus* sp.; yellow box, *Chlamydomonas reinhardtii*; blue star, *Arabidopsis thaliana*. Different colored circles represent different bootstrap values.

We constructed the phylogenetic tree by taking the protein sequences of CaMs/CMLs from *A. thaliana*, *Chlamydomonas reinhardtii*, *S. japonica*, and *E.* sp. (Table S4), and showed three monophyletic groups in the tree (Figure 2). Group I contained eight SjCMLs, eight EsCMLs, and all CaMs. Group III comprised twelve CMLs and three of them were EsCMLs. No brown algae CaMs/CMLs were found in Group II, which only consisted of 32 AtCMLs. In Group I, brown algae CaMs/CMLs (except SjCML7) were assigned in a clade, whereas all AtCaMs were clustered in another clade.

**Figure 3:**
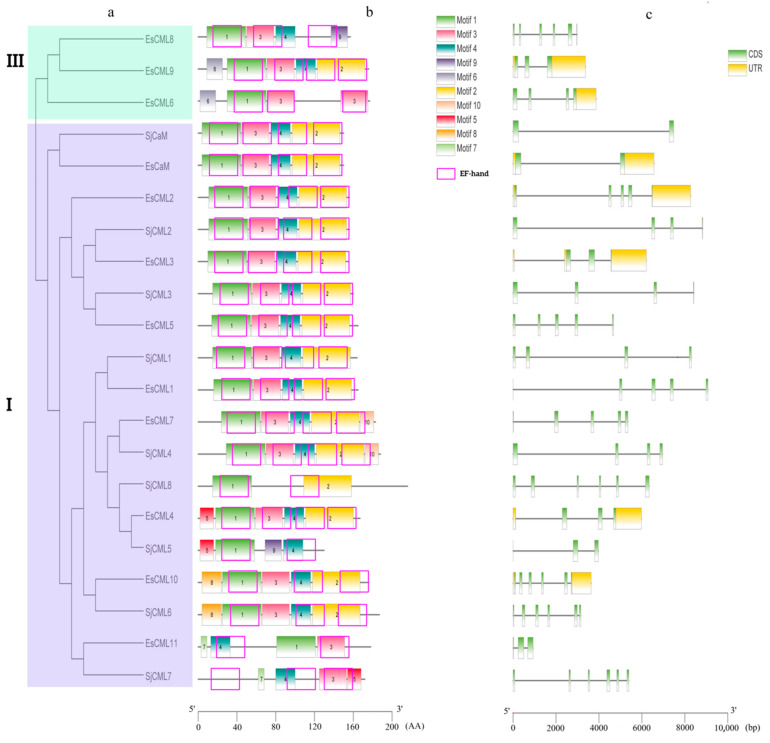
Phylogenetic relationship (**a**), conserved motifs (**b**), and gene structure (**c**) of the brown algae CaMs/CMLs. a: Groups I and III indicate the classification of the brown algae CaMs/CMLs according to the phylogenetic relationship by ML method. b: Motif analysis was performed by the MEME program online. Different colors of boxes represent different motifs in the corresponding position of each CaMs/CMLs protein. EF-hands are marked with pink border rectangle. c: exons and introns are represented by green boxes and the black lines, respectively.

We further comprehensively analyzed the phylogeny, conserved motifs, and gene structures of brown algae CaMs/CMLs (Figure 3 and Table S5). In cases where no full-length cDNA was available, we used partial cDNA (SjCML3 and SjCML5). As shown in Figure 2, 18 brown algae CaMs/CMLs were clustered in one Group (I) and three EsCMLs were clustered in another Group (III). In Group I, CaMs and CMLs were divided into two distinct clades, respectively. CaM genes only contained one intron, which is the same as CaM genes in other plants. Contrastingly, CML genes were intron-rich. The CMLs clade was divided into two subclades. CML genes in one subclade (SjCML2, SjCML3, EsCML2, EsCML3, and EsCML5) and CaMs genes shared the same motif number and similar motif arrangements. CMLs genes in another subclade showed more diversity in the numbers and the structure of introns and motifs.

### 2.3. Chromosomal Distribution and Duplication Analysis of Brown Algae CaMs/CMLs

The nine SjCaM/CML genes were distributed on seven chromosomes in the genome. Both chromosome 0 and chromosome 12 contained two SjCMLs, and the other five chromosomes contained only one SjCaM/SjCML, respectively (Figure S1). The distance between two SjCML genes on the same chromosome was far >150 kb, which indicated that the tandem duplication region was not formed among the SjCaM/CML genes in this study. Meanwhile, the segmental duplication event was not detected using the MCScanX method with E-value < 1 × 10^−10^. The twelve *EsCaM*/*EsCML* genes were distributed on eight chromosomes, and we also did not detect tandem duplication and segmental duplication in *E.* sp.

### 2.4. Molecular Evolution of Brown Algae CaMs/CMLs

To assess the selection pressure of CaMs/CMLs in brown algae, we used the CODEML of the PAML package to compute the dN, dS, and ω values. We found that the ω value of all the brown algae CaMs/CMLs genes was <1 and very low, thus indicating that these genes underwent strong purifying selection (Figure S2). Although ω did not differ significantly among Groups I-CaM, I-CML, and III, the average ω and dN values in Groups I and III (CML genes) were higher than those in Group I (CaM genes), thus suggesting that CMLs may have more extensive functional diversity than CaMs in brown algae.

### 2.5. Cis-Element Analysis of Brown Algae CaMs/CMLs

**Figure 4:**
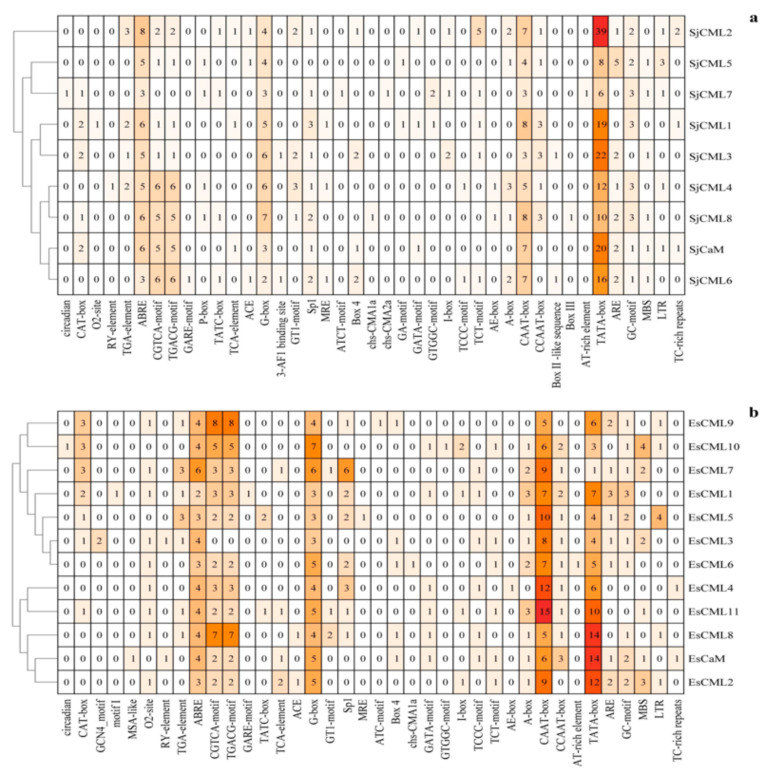
Analysis of the *cis*-acting elements of SjCaM/SjCMLs (**a**) and EsCaM/EsCMLs (**b**). The gradient colors in the orange grid represent the number of *cis*-acting elements in CaMs/CMLs.

We explored the 2 kb upstream region of the start codon ATG of the SjCaM/SjCML and EsCaM/EsCML genes obtained from the genomic sequence. We identified 41 and 38 *cis*-acting elements in promoter regions of SjCaM/SjCML and EsCaM/EsCML genes, respectively (Figure 4 and Table S6). Among them, the ABRE motif, G-box motif, CAAT-box motif, and TATA-box motif were ubiquitously detected in all brown algae CaM/CML genes, thereby indicating their potential critical roles in ABA responsiveness, light responsiveness, and controlling the transcription initiation. Additionally, the CGTCA-motif and TGACG-motif involved in MeJA responsiveness and the stress-related GC-motif involved in anoxic-specific inducibility were also widely presented in both the SjCaM/SjCML and EsCaM/EsCML genes.

### 2.6. Expression Analysis of SjCaM/SjCML Genes

**Figure 5:**
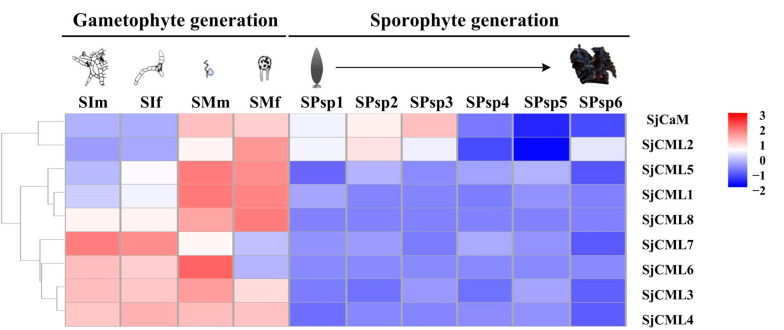
Expression patterns of SjCaM/SjCMLs during different life cycle stages. Log_2_-transformed values to generate heatmaps. SIf: immature female gametophytes; SIm: immature male gametophytes; SMf: mature female gametophytes; SMm: mature male gametophytes. SPsp1-SPsp6: six sporophyte developmental stages.

We evaluated the expression profiles of all the SjCaM/SjCML genes during different life cycle stages using the RNA-sequencing data (Figure 5). In the gametophytic generation, SjCaM/SjCML genes showed variable expression patterns with the gametophyte development. In the mature gametophyte stage, the expressions of SjCaM and four SjCMLs (SjCML1, SjCML2, SjCML5, and SjCML8) were upregulated, whereas that of SjCML7 was downregulated. In contrast, the SjCML6 expression was related to the sexes of gametophyte and was higher in the mature stage than in the immature stage for male gametophytes and vice versa for female gametophytes. There was no significant difference in expression for the remaining two SjCML genes (SjCML3 and SjCML4). Additionally, two SjCML genes (SjCML3 and SjCML6) showed male-biased expression during the mature stage.

As compared to the gametophytic generation, most genes were expressed at lower levels in the sporophytic generation, except SjCaM and SjCML2. Although the expressions of five SjCML genes (SjCML1, SjCML3, SjCML4, SjCML5, and SjCML7) showed fluctuations during the sporophyte development process, the difference was not significant in pairwise comparisons between the six developmental stages. Notably, the expression of two SjCML genes (SjCML6 and SjCML8) could not be detected in any sporophyte developmental stages. Contrastingly, SjCaM was highly expressed in the SPsp1-Spsp3 stages followed by a downregulation in the SPsp4-Spsp6 stages. The SjCML2 expression pattern shared similarity with that of SjCaM, besides its upregulated expression during the SPsp6 stage.

To further verify the expression patterns of these SjCaM/SjCMLs genes, we selected six genes with significant differences during gametophyte development for qRT-PCR analysis (Table S7). The data of these genes were highly consistent with the RNA-seq data (Figure S3). Therefore, these results further suggested that SjCaM/SjCMLs were involved in gametophyte development and could be considered as candidates for further studying their function in *S. japonica*.

### 2.7. Interaction Network Analysis of SjCaM/CMLs

**Figure 6:**
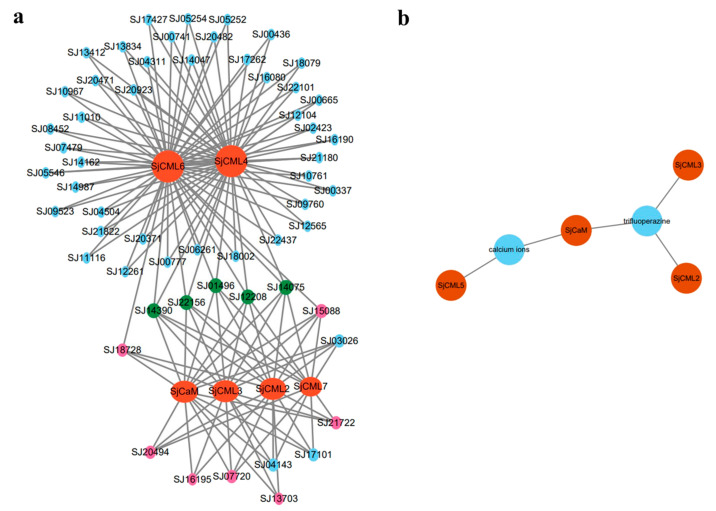
Interaction analysis of SjCaM/SjCMLs using the STRING and STITCH servers. The networks (**a**) show the protein–protein interactions. The red circle identifies SjCaM/SjCMLs, the blue circle identifies SjCaM/SjCMLs interacting proteins, the pink circle identifies calcineurin-like phosphoesterases, the green circle identifies five proteins interacting with all six SjCaM/SjCMLs. The networks (**b**) show the protein–chemical interactions. The red circle identifies SjCaM/SjCMLs and the bule circle identifies predicted interacting chemicals.

We constructed the protein interaction network referring to *Ectocarpus* orthologous proteins to speculate the relationship and synergy between SjCaM/SjCMLs and other *S. japonica* proteins. A total of six SjCaM/SjCMLs interacted with 57 proteins, while three SjCMLs (SjCML1, SjCML5, and SjCML8) did not interact with any proteins (Figure 6a). Among these interacting proteins, calcineurin-like phosphoesterases were the most abundant (Table S8). In addition, five proteins were predicted to interact with all six SjCaM/SjCMLs, including a protein kinase (SJ01496), a cytoskeletal adhesion protein (SJ14075), a tubulin protein (SJ22156), a SAC3/GANP protein (SJ12208), and a hypothetical protein (SJ14390) (Figure 6a). GO functional enrichment analysis showed that a total of 151 GO terms enriched for SjCaM/SjCMLs interacting proteins, including 113 (74.8%) biological process terms (BP), 25 (16.6%) molecular function terms (MF), and 13 (8.6%) cellular component terms (CC). The interacting proteins were significantly enriched in “cellular process” and “response to stimulus” in the BP category, “cell” and “cell part” in the MF category, and “non-membrane-bounded organelle” in the CC category (Table S9). The KEGG pathway analysis revealed that four pathways, including “Nucleotide excision repair”, “Phagosome”, “mRNA surveillance pathway”, and “Mismatch repair”, were significantly enriched (Figure S4).

Due to the lack of protein–chemical interaction information on brown algae in the STITCH database, we constructed the protein–chemical interaction network referring to the organism of *A. thaliana*. The result suggested the interaction of four SjCaM/CMLs with two different chemicals: trifluoperazine and calcium ions (Figure 6b). Among them, the SjCaM protein interacted with both chemicals. The two proteins SjCML2 and SjCML3 showed interaction with trifluoperazine, while the SjCML5 protein interacted with calcium ions.

## 3. Discussion

As Ca^2+^ sensors, CaM and CML genes are widely involved in plant growth and development and also the mediation of plant stress tolerance. However, whole-genome-scale studies about CaM/CML families have not been performed in brown algae, which have evolved independently of the well-studied green plant lineage. In this study, we identified one SjCaM/8 SjCMLs and one EsCaM/11 EsCMLs in *S. japonica* and *E.* sp., respectively. The size of the CaM/CML families in brown algae was smaller than those in the plant species, but showed similarity with green algae, including *Klebsormidium flaccidum* (one CaM and eight CMLs) and *Chlamydomonas reinhardtii* (one CaM and nine CMLs) [12]. In the green lineage, two major transitions in the evolution of CaM and CML gene numbers from green algae to angiosperm were likely associated with the acquisition of multicellularity, the adaption of terrestrial environments, and the development of novel functions necessary for colonizing new habitats. In brown algae, *Ectocarpus* is multicellular single-row filamentous, whereas the *Saccharina* sporophyte showed a morphological differentiation of rhizoids, stipes, and blades. Nonetheless, a limited number of *CaM* and *CML* genes were found in these two marine brown algae species, thus indicating that the acquisition of novel tissue differentiation-related functions could not be the evolutionary driving force of the expansion of *CaM*/*CMLs* in brown algae.

We constructed an ML phylogenetic tree based on amino acid sequences from brown algae (*S. japonica* and *E.* sp.) and green lineage species (*C. reinhardtii* and *A. thaliana*) to determine the phylogenetic relationship and further validate the identification of brown algae CaM/CMLs. As shown in Figure 2, all the CaMs were assigned to Group I. SjCaM and EsCaM were closer to AtCaMs than CrCaM, with CrCaM forming the basal root. This result indicates that plant and brown algae CaMs evolved from the common ancestor of basal unicellular eukaryotic lineages, which may have originated from the bacterial endosymbiotic event due to the presence of EF-hand-containing proteins in many bacteria and the widespread presence of CaMs among eukaryotes [12,52]. In Group I, most brown algae CaMs/CMLs were clustered in a different clade to AtCaMs, indicating the independent evolution of brown algae CaMs/CMLs. Additionally, most AtCMLs were clustered in Group II, in which no brown algae CMLs and green algae CMLs were found, thus illustrating that these AtCMLs might have evolved by duplication and acquired their diverse functions to respond to environmental signals as plants have adapted to their terrestrial environments.

In this study, we identified only one SjCaM and one EsCaM. They encoded identical protein products and showed high degrees of amino acid identities (88.59–89.93%) and typical gene structure characteristics with AtCaMs, including lysine at position 116, six conserved amino acids in the Ca^2+^ binding loop, the distribution pattern of hydrophobic amino acids in both the E and F helices, a high proportion of methionine, four EF-hand motifs, and one intron. The highly conserved structure of CaMs shared between *S. japonica/E.* sp. and *A. thaliana* indicates that brown algae CaMs may retain the similar function of being Ca^2+^ sensors in cell signaling, like in plants. In contrast, CMLs showed greater diversity in length, gene structure, methionine percentage, and EF-hand number in brown algae. The amino acid identity ranged from 19.88% to 61.94% among the SjCMLs (Table S10). However, the tandem duplication and segmental duplication events were not detected in brown algae CaM/CMLs. The molecular evolution analysis showed brown alga CMLs had undergone strong purifying selection during evolution, thus indicating that the function of brown alga CMLs may be relatively conserved, and the functional differentiation is limited. This may be related to the fact that brown algae do not need to face complex environments like terrestrial plants.

CaM and CML genes play diverse and multiple roles in plant growth, development, and stress responses. To explore the potential functions of brown algae CaMs/CMLs, we first analyzed the *cis*-acting elements due to their key role in gene expression regulation. The results showed that “light response elements” had the highest abundance of *cis*-acting elements in both SjCaM/SjCMLs (41.5%) and EsCaM/EsCMLs (36.8%), with all the brown algae CaMs/CMLs identified in this study containing the light-responsiveness-related element (G-box motif). This result indicates that CaM/CMLs may be vital in the growth and developmental processes of brown algae. Additionally, the ABRE motif was also ubiquitous in the brown algae CaMs/CMLs. ABRE is a key *cis*-regulatory element involved in ABA signaling and mediates plant response to many abiotic stresses, including osmotic, drought, heat, and cold [53,54]. Thus, we speculated that CaM/CML genes may be involved in the mediation of ABA signaling in the abiotic stress response of brown algae. Then, we analyzed the expression patterns of SjCaM/SjCML genes in different development stages of two life cycle generations. Generally, the expressions of SjCaM/SjCML genes in gametophyte generation were greater than those in sporophyte generation, thus suggesting they may be mainly involved in the regulation of gametophyte developmental. SjCaM/SjCML genes showed various expression patterns during gametophytic development. For example, one SjCaM gene and four SjCML genes were induced and one SjCML7 gene was repressed in mature gametophytes, thus indicating that these genes could participate in gametophyte growth and development by *cis*-regulation and trans-regulation, respectively. The expressions of SjCML3 and SjCML6 were upregulated significantly only in the mature male gametophytes. The male-biased expression in mature gametophytes indicated these two genes may be mainly involved in spermatogenesis or sperm development. Although the expression of CaM/CML genes during fertilization, zygotic, and early embryonic development was not explored in this study, CaM’s involvement in these developmental processes in brown algae has been reported in previous studies [36,37,38]. Based on the culture conditions of *S. japonica* gametophytes and the *cis*-acting element characteristics of SjCaM/SjCML genes, we discussed the factors that induced gene expression changes. *S. japonica* gametophytes are microscopic, filamentous, and dioicous. The gametophyte generation has a very short life cycle as it takes only ~20 days from gametophyte formation to the release of gametes through gametogenesis in natural conditions. To preserve gametophytes in the lab for a long time, we cultured the immature gametophytes under vegetable growth conditions, which is regarded as the adaptive form for stressful environments [55]. Light intensity, photoperiod, and nutrient status were important conditions for the initiation of gametogenesis and the maturation of gametophytes in brown algae, including kelp [56,57,58,59]. Therefore, we adjusted the conditions of the light intensity, photoperiod, and culture medium to obtain mature gametophytes in this study. Meanwhile, many light-responsive elements (e.g., G-box motif) were ubiquitous in the upstream region of the identified brown algae CaM/CML genes, thus indicating that these genes directly responded to light-related stress recovery to be involved in the pathway of gametophytes maturation. Additionally, hormone-responsive elements, including ABRE, CGTCA-motif, and TGACG-motif, were also widespread in the promoters of the brown algae CaM/CML genes. This indicates that these genes may be indirectly involved in stress response via the hormone regulation pathway. Under external stimuli, cytosolic Ca^2+^ concentration increased through either the influx of extracellular Ca^2+^ or the temporary release of intracellular stores. Ca^2+^ binding proteins decoded Ca^2+^ signals and regulated the corresponding physiological processes subsequently. A calcium influx induced by gamete fusion played a direct role in egg activation during the development of brown algae [60]. This indicates that the influx of calcium ions in the culture medium via the Ca^2+^ channel may have led to changes in the expression of CaM and CMLs during gametophyte development in this study. However, we cannot rule out the mobilization of intracellular Ca^2+^ from storage compartments, such as the vacuole and the endoplasmic reticulum.

The sporophytes we collected from January to July in this study should have faced variable environmental conditions. Unexpectedly, the expressions of most SjCML genes were very low and did not change significantly during the generation of sporophytes. The differential expression detected in the SjCaM and SjCML2 genes may be related to the reduced growth rate at the SJsp4 stage and the appearance of sporangia on the sporophyte blade at the SJsp6 stage [61].

We predicted a total of 57 interacting proteins using high-confidence criteria (interaction score ≥0.70) with STRING. The protein interaction network showed that calcineurin-like phosphoesterases were the most abundant. The calcineurin-like phosphoesterases family includes a diverse range of phosphoesterases, which are related to the calcium- and calmodulin-dependent serine/threonine protein phosphatase. They could be involved in signal transduction related to various stress responses in plants, such as *p* starvation, drought stress, and zine exposure [62,63,64]. There were five proteins predicted to interact with all six SjCaM/SjCMLs. SJ01496 encodes a protein kinase that contains an STKc_CAMK domain. It may be a key player in the Ca^2+^/CaM-mediated signaling pathway by catalyzing protein phosphorylationas as a calmodulin-binding protein kinase [65,66]. SJ14075 and SJ22156 encode a cytoskeletal adhesion protein and a tubulin protein, respectively. They may play a role in the regulation of microtubule cytoskeleton in growth and development as CaM-binding microtubule-associated proteins [67]. SJ12208 was predicted to be a SAC3/GANP family protein. A recent experiment demonstrated that CML19 could interact with SAC3B to form the transcription export complexes in *A. thaliana* [68]. These genes could be used as candidates for studying the regulatory network of CaM/CMLs. The protein–chemical interaction analysis has been used to understand the molecular and cellular function of proteins [69,70]. In this study, the interaction of SjCaM/CMLs with trifluoperazine and calcium ions indicated their probable role in Ca^2+^ signaling. Trifluoperazine is a well-known CaM antagonist that can bind to CaM and modulate the interactions between CaMs and their target biological processes [71,72]. The protein–protein and protein–chemical interactions found in this study need to be validated in future studies in brown algae.

## 4. Materials and Methods

### 4.1. Identification and Characterization of CaM/CML Genes in S. japonica and E. sp.

The genome and protein sequences of brown algae (*S. japonica* and *E. siliculosus* V2) were downloaded from ORCAE (https://bioinformatics.psb.ugent.be/orcae/, accessed on 10 September 2022). The protein sequences of CaMs/CMLs in *A. thaliana* [11,73] and *O. sativa* [13] were downloaded from TAIR (http://www.arabidopsis.org, accessed on 10 September 2022) and RAP-DB (https://rapdb.dna.affrc.go.jp/, accessed on 10 September 2022), respectively, and used as queries (Blastp, E-value < 1×10^−5^) to search against the brown algae genome database. The obtained sequences from *S. japonica* and *E.* sp. were further used to construct the hidden Markov model (HMM) profile using HMMER 3.3.2 (http://hmmer.org/download.html, accessed on 11 September 2022), which was then searched against the local protein database of each species. Based on the results of BLAST hits and HMMER, the common sequences of each species were selected and manually examined for the EF-hand domain using the Pfam/NCBI/SMART database. Those proteins containing only the EF-hand functional domain and <400 amino acids were considered putative SjCaM/SjCML and EsCaM/EsCML proteins for *S. japonica* and *E.* sp., respectively. The theoretical isoelectric point (pI) and molecular weight (Mw) of each CaM/CML protein was calculated using ExPASy (https://web.expasy.org/compute_pi/, accessed on 15 September 2022). Subcellular localization of each SjCaM/SjCML and EsCaM/EsCML protein was predicted by the WoLF PSORT tool (https://www.genscript.com/wolf-psort.html, accessed on 19 April 2023).

### 4.2. Alignment and Phylogenetic Analysis of Brown Algae CaM/CML Genes

The sequences of CaM/CML proteins of *C. reinhardtii* were obtained from Phytozome (https://phytozome-next.jgi.doe.gov/, accessed on 25 September 2022). Alignments were performed by the multiple sequence alignment program ClustalW using default settings. The alignment of CaM proteins was viewed using SeqVu1.0.1 (Garvan Institute of Medical Research, Sydney, Australia). The Maximum-Likelihood (ML) phylogenetic tree was constructed using ATGC PhyML 3.0 with 1000 bootstrap replicates (http://www.atgc-montpellier.fr/phyml/, accessed on 15 October 2022) [74] and was displayed using Evolview: Tree View (http://www.evolgenius.info/evolview/, accessed on 17 October 2022) [75].

### 4.3. Gene Structure and Cis-Acting Elements Analysis of Brown Algae CaM/CML Genes

Structure information on the SjCaM/SjCML and EsCaM/EsCML genes was obtained from their respective GFF files. The conserved motifs of CaMs/CMLs were analyzed using the MEME program (https://meme-suite.org/meme/tools/meme, accessed on 20 October 2022) with a motif number setting of 10 [76]. Gene structures and motifs were visualized using the TBtools software (https://github.com/CJ-Chen/TBtools/releases, accessed on 8 March 2023; Version number: v1.108; Creator: Chen Chengjie; Guangzhou, China) [77]. To assess the regulatory elements of brown algae CaM/CML genes, 2000 bp upstream of the coding sequences of SjCaM/SjCML and EsCaM/EsCML genes were extracted for the *cis*-acting element analysis using the PlantCARE database (https://bioinformatics.psb.ugent.be/webtools/plantcare/html/, accessed on 25 October 2022) [78].

### 4.4. Chromosomal Distribution, Gene Duplication, and Selection Pressure of Brown Algae CaM/CML Genes

The chromosomal location information on the brown algae CaM/CML genes was also obtained from the GFF file, and the chromosomal distribution of SjCaM/SjCMLs and EsCaM/EsCMLs was presented using the TBtools software [77]. The duplication events of SjCaM/CMLs were analyzed by MCScanX program in TBtools software. To evaluate the variation in selective pressures for each CaM/CML gene or groups of CaM/CML genes, the nonsynonymous substitution values (dN), synonymous substitution values (dS), and nonsynonymous/synonymous substitution rate (ω = dN/dS) were calculated using the CODEML program in the PAML software package (http://abacus.gene.ucl.ac.uk/software/paml.html, accessed on 8 March 2023; Version number: 4.9j; Creator: Yang Ziheng; London, UK) [79].

### 4.5. Expression Analysis of the SjCaM/SjCMLs in Different Life Cycle Stages

To gain insight into the expression characteristics of the SjCaM/SjCML genes at different developmental stages, the transcriptome sequencing data were obtained from our previous studies under the accession numbers PRJNA656182 and PRJNA511944, which represented the gametophyte and sporophyte generation, respectively [45,61]. Two male gametophyte developmental stages (SIm and SMm), two female gametophyte developmental stages (SIf and SMf), and six sporophyte developmental stages (SJsp1–SJsp6) were subjected to RNA-sequencing. Three biological replicates were tested for each condition. The FPKM value (Fragments Per Kilobase of transcript per Million fragments mapped) of each SjCaM/SjCML gene was calculated and normalized using the log_2_-transformed values to generate heatmaps. Differential gene expression levels among the different samples were analyzed based on an absolute log_2_ (fold change) > 1 and adjusted *p*-value < 0.05.

### 4.6. qRT-PCR Analysis

The six differentially expressed SjCaM/SjCML genes involved in gametophyte generation were selected for qRT-PCR analysis. The immature gametophytes were preserved in seawater (supplemented with 12 μmol NaNO_3_ and 7.35 μmol KH_2_PO_4_) at 10 °C under a constant irradiance of 15–20 μmol photos m^−2^ s^−1^. The mature gametophytes were collected after being cultured at 10 °C in the Provasoli-enriched natural seawater [80] under 40–50 μmol photons m^−2^ s^−1^ with a photoperiod of 12:12 h (light/dark). Total RNA was extracted using the RNeasy Plant Mini Kit (Qiagen, Hilden, Germany) according to the manufacturer’s procedure. The cDNA was synthesized using the PrimeScript RT Reagent Kit with gDNA Eraser (Takara, Dalian, China). The expression profile of the SjCaM/SjCML genes was examined using SYBR Green PCR Master Mix (Takara, China). The EF1α was selected as the internal control and the 2^−ΔΔCt^ method was used to assess the expression level of genes [81]. The expression levels were compared using *t*-test (*p* < 0.05) in the SPSS 19.0 software (https://www.ibm.com/spss, accessed on 8 March 2023; Version number: 19.0; New York, NY, USA).

### 4.7. Protein–Protein and Protein–Chemical Analysis

The CaMs/CMLs protein–protein and protein–chemical interactions were performed using the STRING (https://cn.string-db.org/, accessed on 10 December 2022) and STITCH (http://stitch.embl.de/, accessed on 21 April 2023) servers, respectively. Protein interaction network was constructed with the reference organism of *E. siliculosus*. Protein–chemical interaction network was constructed with the reference organism of *Arabidopsis thaliana*. Proteins or chemicals with a combined interaction score ≥ 0.70 were selected. The network was visualized using Cytoscape (version 3.9.1). Gene Ontology (GO) and Kyoto Encyclopedia of Genes and Genomes (KEGG) analysis of interaction proteins were performed the TBtools software [77].

## 5. Conclusions

This study was the first to systematically identify *CaM* and *CML* genes in brown algae. There were one SjCaM/eight SjCMLs and one EsCaM/eleven EsCMLs found in *S. japonica* and *E.* sp., respectively. The phylogenetics, gene structures, gene duplication, and molecular evolution analysis helped investigate their evolutionary history. According to *cis*-acting regulatory element and expression analysis, CaM/CMLs might be involved in the development of and stress response in brown algae, and their expression patterns in gametophyte development were also validated in *S. japonica* by qRT-PCR. The interaction analysis suggested the involvement of *SjCaM/CML* genes in Ca^2+^/CaM-mediated developmental processes and stress responses and provided the candidates for studying the CaM/CMLs regulatory network. This study indicates that SjCaM/SjCMLs have diverse functions in gametophyte development, but the specific role of each gene needs to be further determined in future research.

## Figures and Tables

**Table 1:** Characteristics of CaM/CML proteins in *S. japonica* and *E.* sp.

Name	Gene ID	Length (aa)	EF Hands	Met (%)	Lys (116)	pI	MW (kDa)	Identity to SjCaM/EsCaM	Intron	Subcellular Location
SjCaM	SJ01891	149	4	7.4	+	4.09	16.77	100	1	Cytoplasmic
SjCML1	SJ02978	163	4	4.9		4.66	18.70	54.36	3	Cytoplasmic
SjCML2	SJ02822	155	4	5.2		4.04	17.28	50.68	3	Chloroplast
SjCML3	SJ02784	159	4	6.9		4.19	17.89	45.27	3	Nuclear
SjCML4	SJ15309	187	4	3.7		4.54	20.96	42.28	3	Chloroplast
SjCML5	SJ16374	130	2	5.4		4.85	14.60	38.79	2	Nuclear
SjCML6	SJ16755	186	3	2.2		4.94	20.74	36.91	5	Cytoplasmic
SjCML7	SJ12528	171	3	4.1		6.10	19.51	28.00	5	Nuclear
SjCML8	SJ17363	215	2	6		4.62	23.47	27.52	5	Chloroplast
EsCaM	Ec-06_002270.1	149	4	7.4	+	4.09	16.77	100	1	Cytoplasmic
EsCML1	Ec-15_000530.1	164	4	5.5		4.66	18.86	54.36	4	Cytoplasmic
EsCML2	Ec-24_000510.1	155	4	5.2		4.19	17.40	52.03	4	Cytoplasmic
EsCML3	Ec-23_000190.1	154	4	5.2		4.03	17.13	50.68	2	Nuclear
EsCML4	Ec-04_003890.1	166	4	5.4		4.50	18.93	46.31	3	Mitochondrial
EsCML5	Ec-23_001960.1	164	4	3.7		4.37	18.27	44.3	4	Cytoplasmic
EsCML6	Ec-11_005890.1	176	3	4		4.68	19.74	42.39	3	Peroxisomal
EsCML7	Ec-06_007660.1	182	4	3.8		4.48	20.39	41.61	4	Mitochondrial
EsCML8	Ec-16_003140.1	156	3	5.1		4.06	17.44	38.26	5	Cytoplasmic
EsCML9	Ec-06_003120.1	175	4	3.4		4.58	19.61	36.24	2	Chloroplast
EsCML10	Ec-26_003440.1	175	3	2.9		5.42	19.82	35.57	5	Cytoplasmic
EsCML11	Ec-16_003330.1	177	2	4.5		4.47	19.12	26.03	2	Mitochondrial

aa, amino acids; Met, methionine; Lys, lysine; pI, isoelectric point; MW, molecular weight.

## Data Availability

Not applicable.

## Supplementary Materials

The following supporting information can be downloaded at https://www.mdpi.com/article/10.3390/plants12101934/s1, Figure S1: Distribution of CaM/CML genes in chromosome of *S. japonica* (a) and *E.* sp. (b); Figure S2: Nonsynonymous/synonymous substitution rate (ω) of brown algae CaM/CML genes in different phylogenetic groups; Figure S3: Validation of gene expression by qRT-PCR; Figure S4: KEGG enrichment analysis of SjCaM/SjCMLs interacting proteins; Table S1: List of EF-hand-domain-containing proteins in *S. japonica* and *E.* sp.; Table S2: Protein sequences of brown algae CaMs/CMLs; Table S3: Protein (lower left) and nucleotide (upper right) sequence identity (%) of CaMs among the brown alga and green lineage; Table S4: CaMs and CMLs proteins used for phylogenetic analysis and their group information; Table S5: The amino acid sequence of conserved motifs of CaM and CML proteins; Table S6: *Cis*-acting elements in promoter regions of brown algae CaMs/CMLs; Table S7: List of qRT-PCR primers used in this study; Table S8: List of SjCaM/SjCMLs interacting proteins; Table S9: GO enrichment of SjCaM/SjCMLs interacting proteins; Table S10: Protein (lower left) and nucleotide (upper right) sequence identity (%) of CaM and CMLs in *S. japonica*.
