# Supplementary figures and images for: Genome-Wide Identification and Expression Analysis of Calmodulin (CaM) and Calmodulin-Like (CML) Genes in the Brown Algae Saccharina japonica
Source: Plants (Basel). 2023 May 9;12(10):1934. doi: 10.3390/plants12101934 (PMC10222329; doi:10.3390/plants12101934)

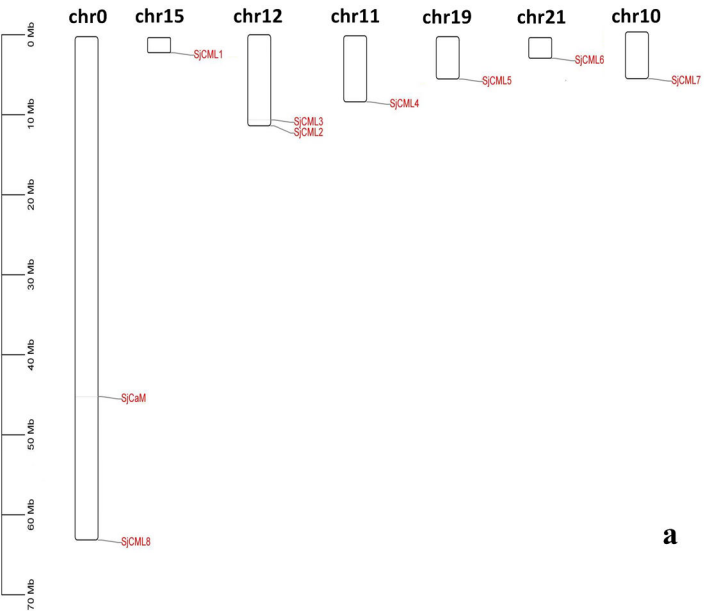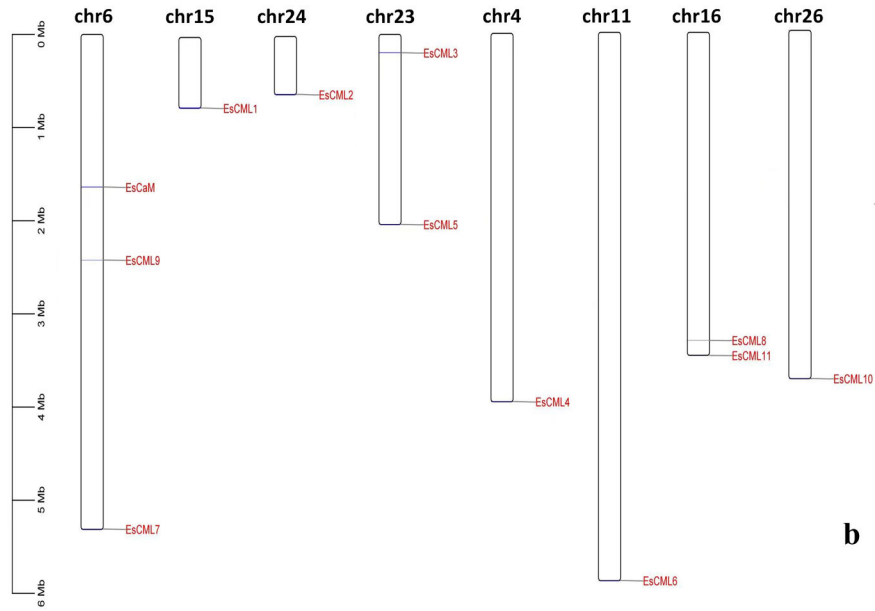

Supplement: Supplementary file 1 [file plants-12-01934-s001.zip › Figure S1 .pdf]

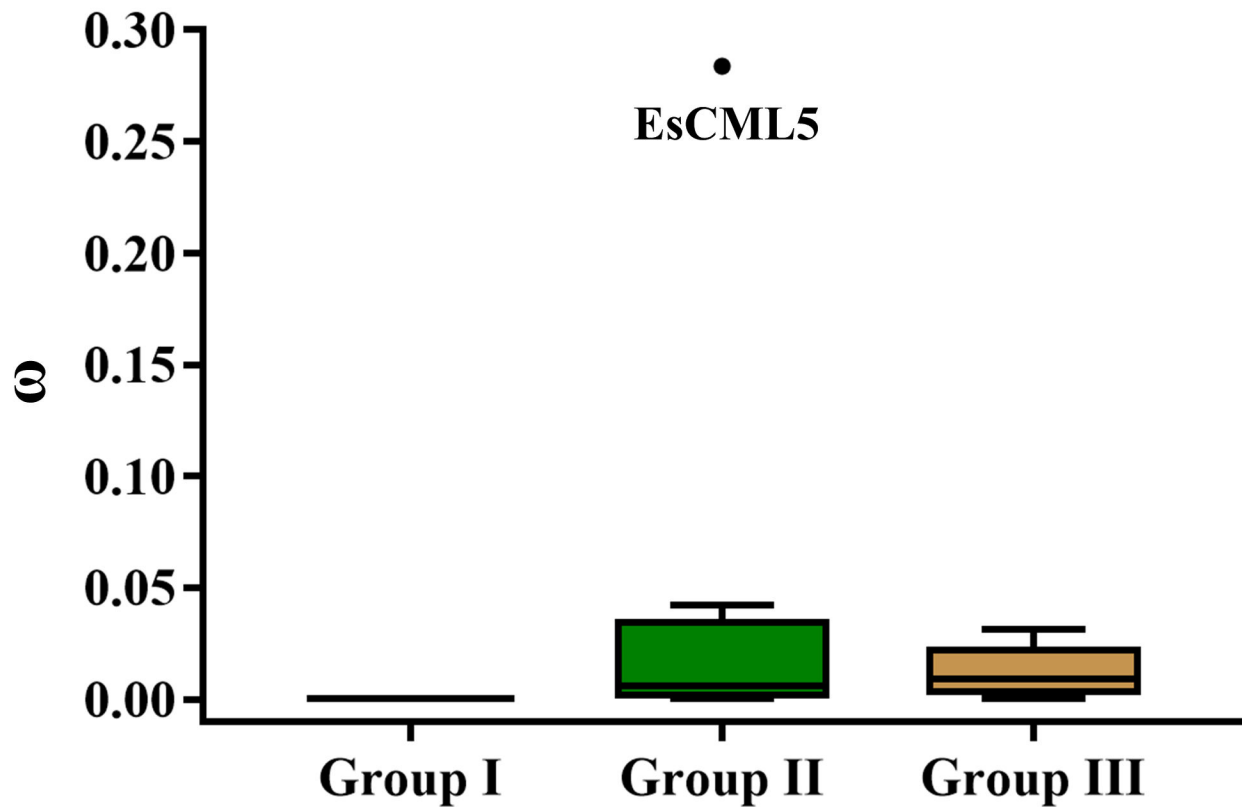

Supplement: Supplementary file 1 [file plants-12-01934-s001.zip › Figure S2.pdf]

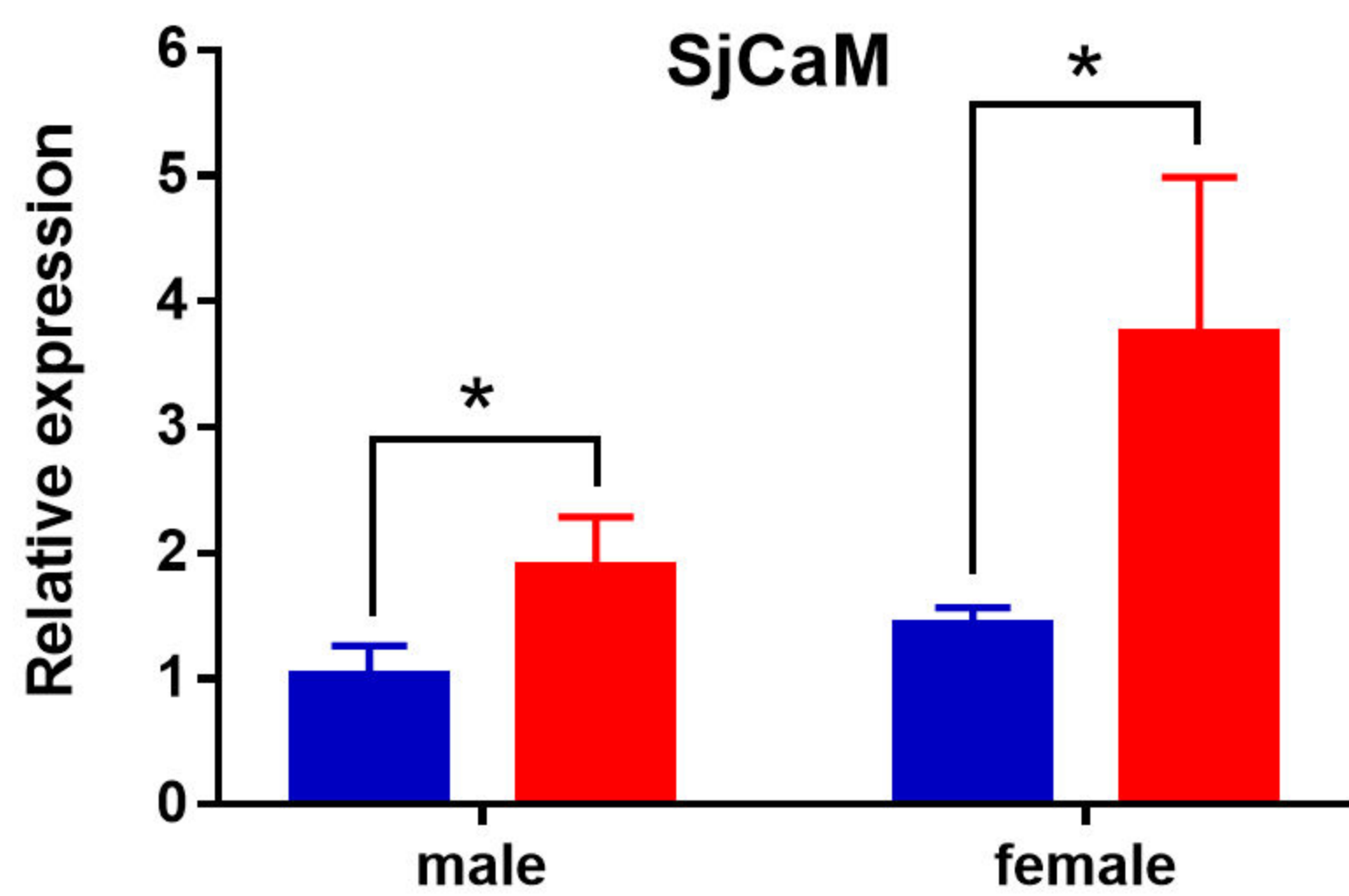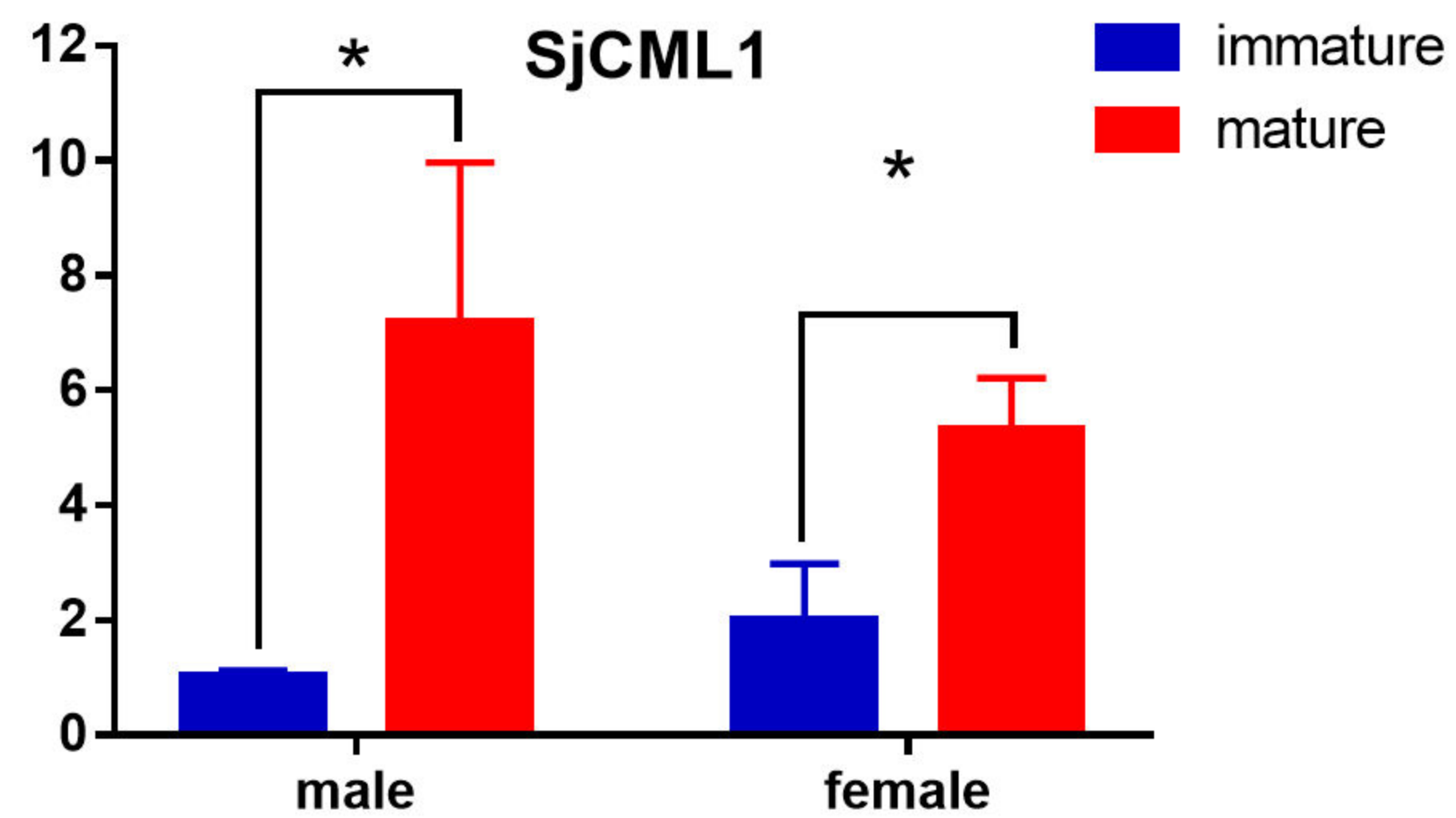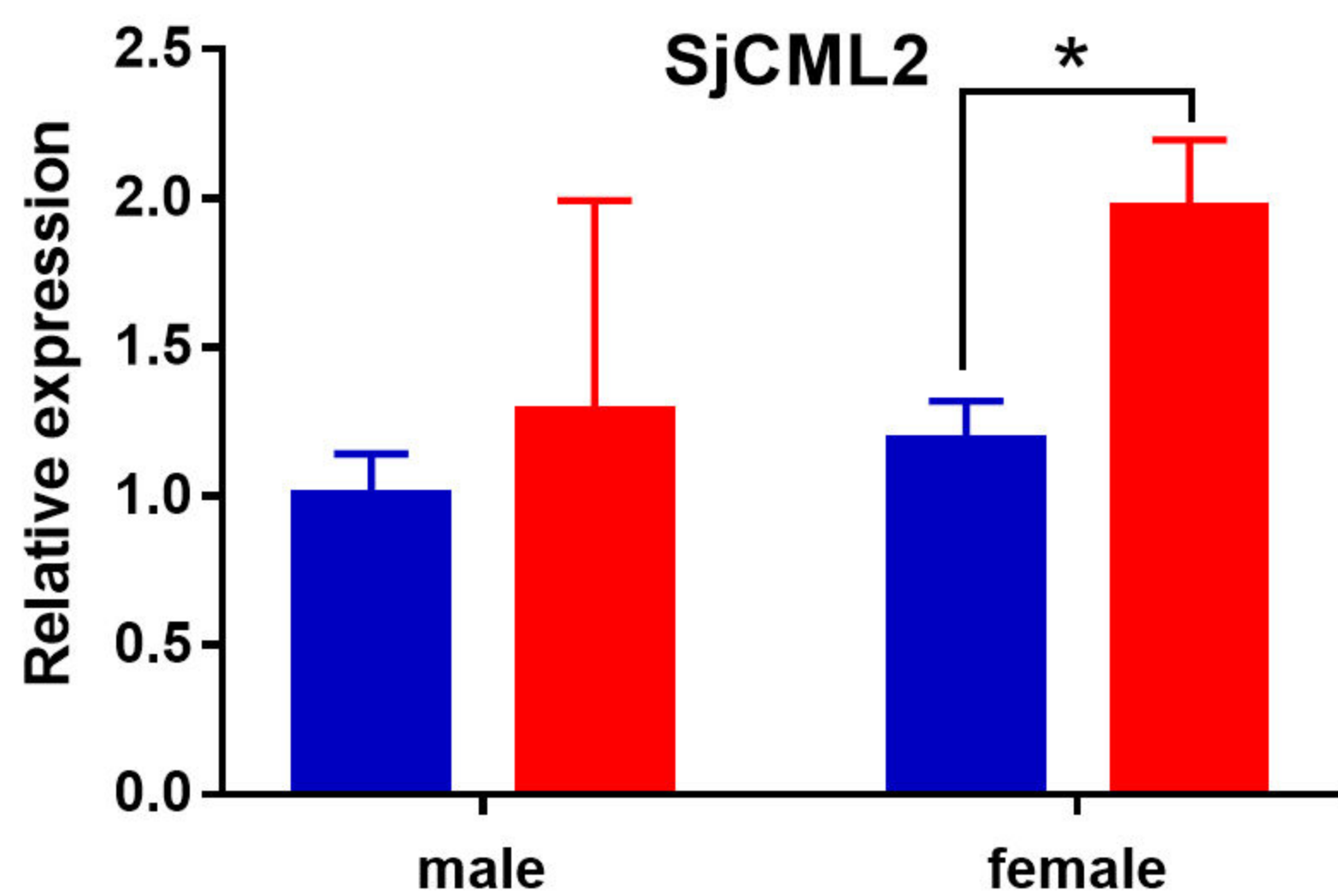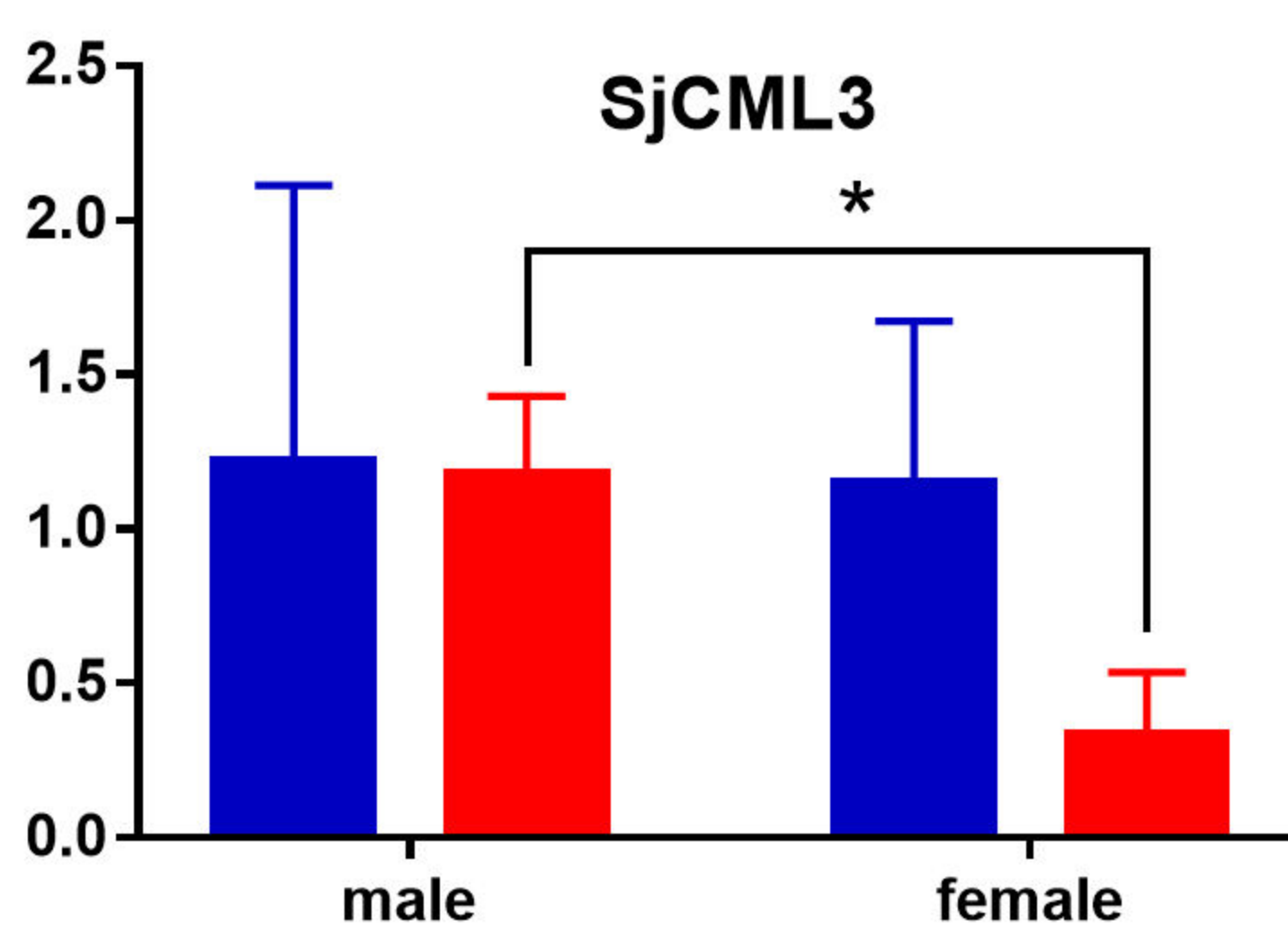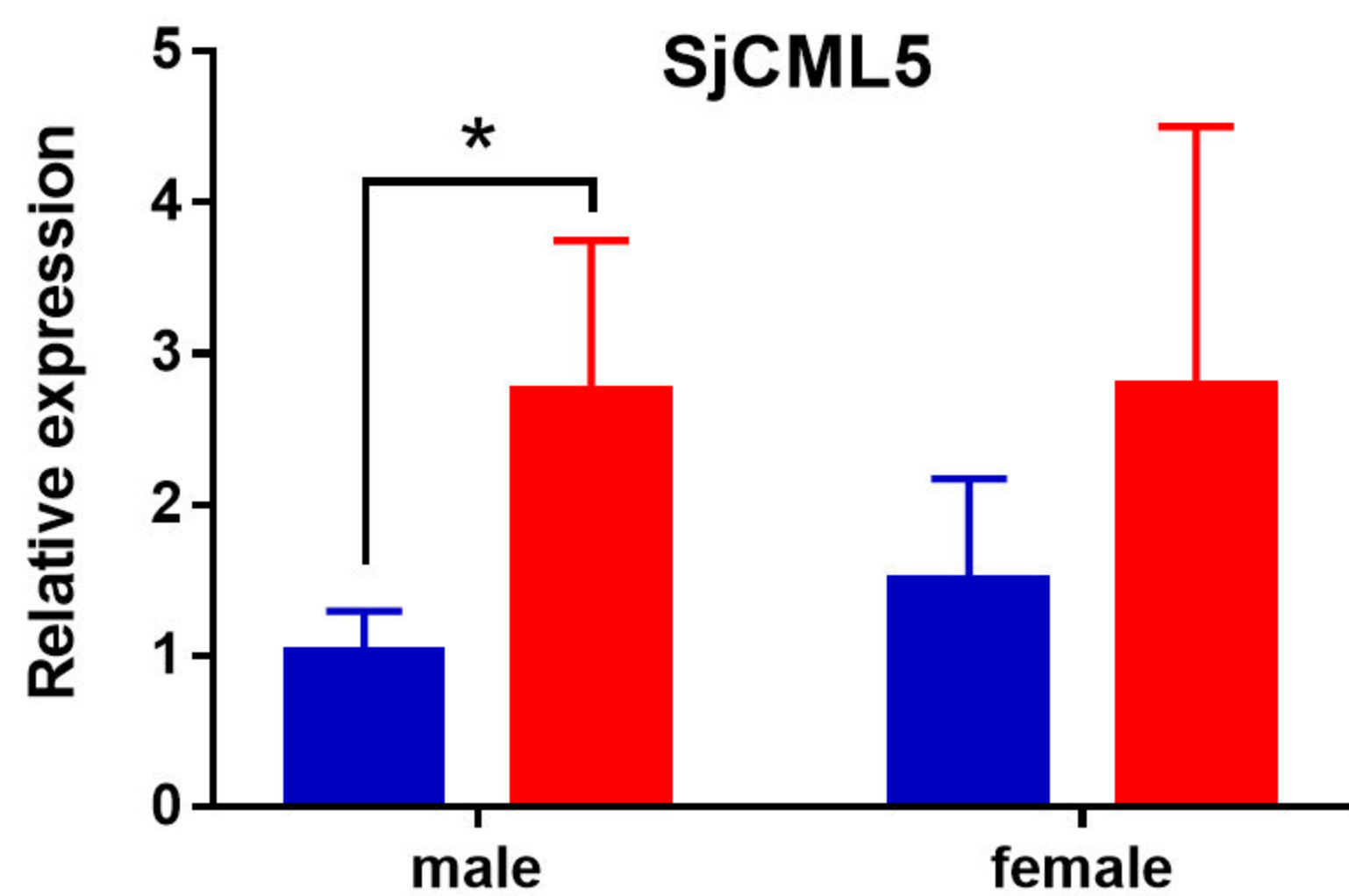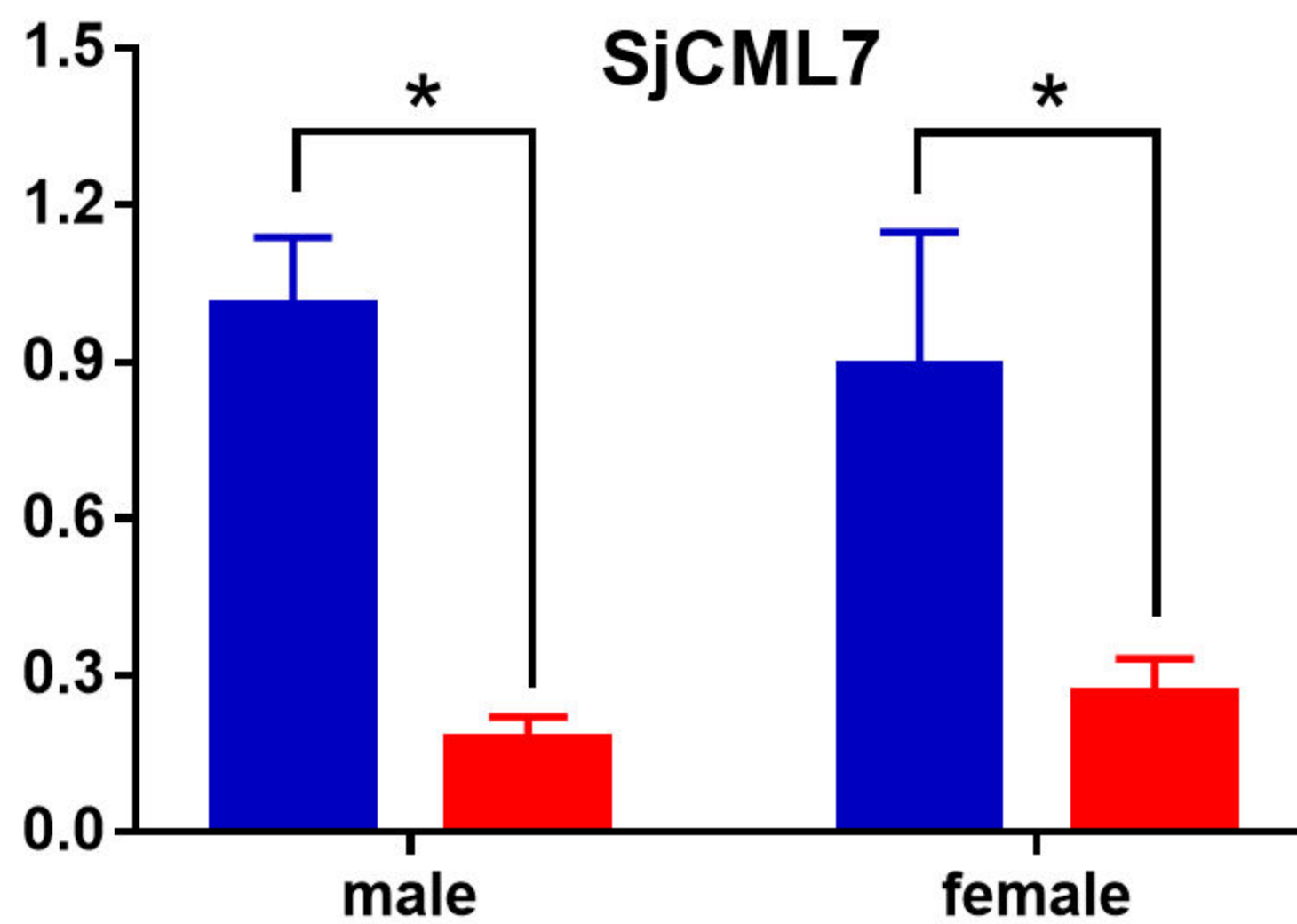

Supplement: Supplementary file 1 [file plants-12-01934-s001.zip › Figure S3.pdf]

Statistics of Pathway Enrichment

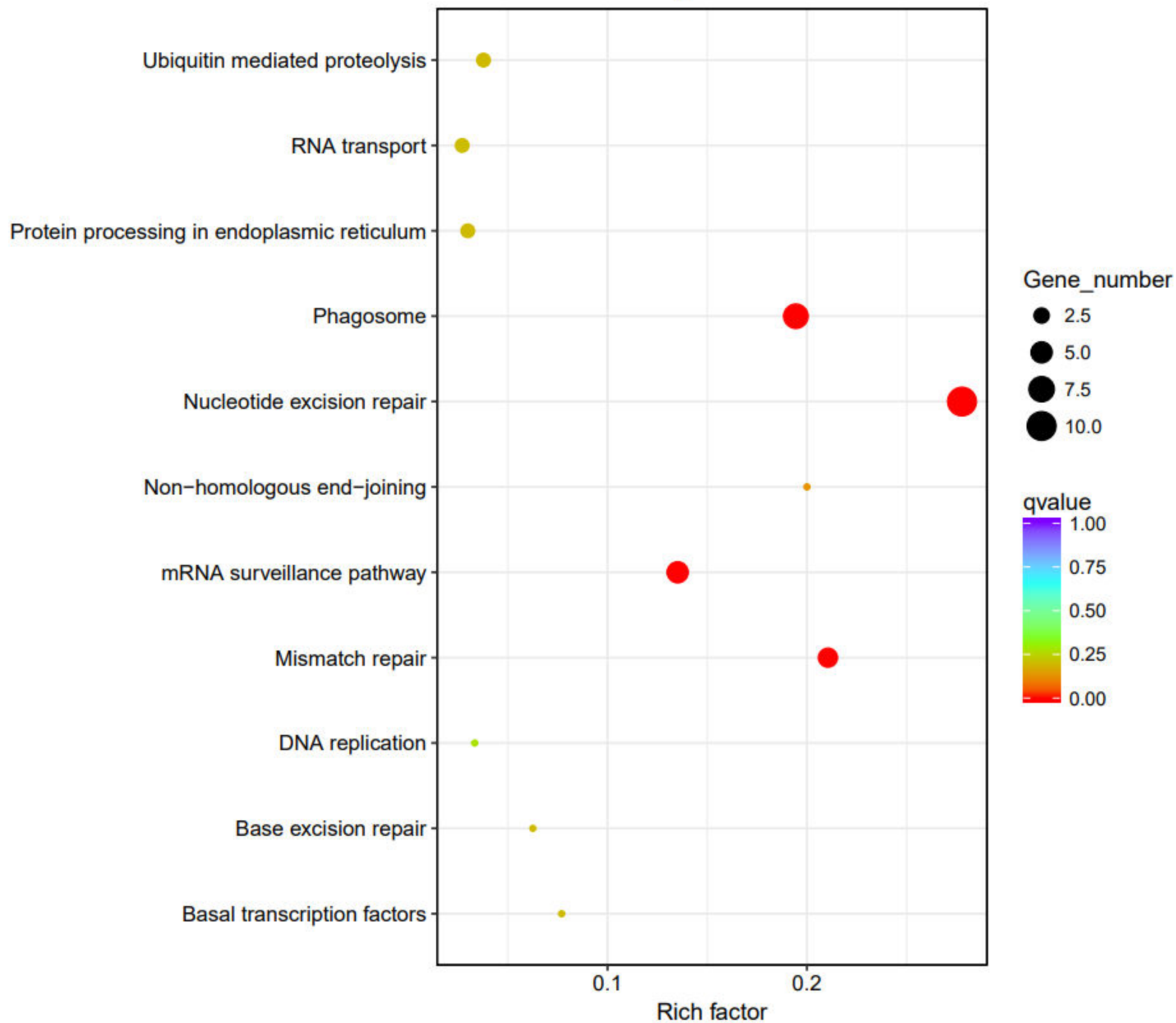

Supplement: Supplementary file 1 [file plants-12-01934-s001.zip › Figure S4.pdf]
